# Protective Effects of Evodiamine against LPS-Induced Acute Kidney Injury through Regulation of ROS-NF-*κ*B-Mediated Inflammation

**DOI:** 10.1155/2019/2190847

**Published:** 2019-03-03

**Authors:** Yan Shi, Qiuju Hua, Na Li, Min Zhao, Yan Cui

**Affiliations:** Hospital of Nephrology, The First Affiliated Hospital of Xinxiang Medical University, Weihui, Henan 453100, China

## Abstract

Acute kidney injury (AKI) is a critical care syndrome, which is usually associated with sepsis-related endotoxemia. Evodiamine (EVO) is an active ingredient of many traditional medicinal formulations that possess a battery of biological activities. In the study, we aimed to evaluate the potential protective effect of EVO against lipopolysaccharide- (LPS-) induced AKI and cytotoxicity. LPS-resulted pathological injuries were significantly ameliorated by the administration of EVO. EVO reduced the levels of blood urea nitrogen (BUN) and creatinine in LPS-treated rats. EVO also inhibited LPS-induced reduction of cell viability in NRK-52E cells. LPS-resulting increase of TNF*α* and IL-1*β* in both serum and kidney of rats and NRK-52E cells was inhibited by EVO. LPS-induced increase of P65 NF-*κ*B expression was markedly inhibited by EVO. EVO-induced reduction of TNF*α* and IL-1*β* expression in LPS-treated cells was blocked by overexpression of P65 NF-*κ*B. Moreover, the increase of cell viability in LPS-treated cells induced by EVO was remarkably suppressed by overexpression of P65 NF-*κ*B. LPS-resulting increase of reactive oxygen species (ROS) production was suppressed by EVO. H_2_O_2_ suppressed EVO-induced decrease of P65 NF-*κ*B expression and increase of cell viability in LPS-treated NRK-52E cells. Moreover, the antioxidant NAC significantly promoted EVO-induced decrease of P65 NF-*κ*B expression and increase of cell viability in LPS-treated NRK-52E cells. In conclusion, EVO had crucial protective effects against LPS-induced AKI and cytotoxicity through the antioxidant activities and thus the inhibition of inflammation. Our data highlight EVO as a potential candidate for the development of new strategies for the treatment of AKI.

## 1. Introduction

Kidney is an important organ functioning to filter blood and acting as the defense line in the body [1]. It is unfortunate that kidney is one of the most common and direct targets of severe damage [2]. Acute kidney injury (AKI) is a critical care syndrome, which is very common in the elderly with up to 22% mortality of hospitalized patients [3–6]. AKI is usually associated with sepsis-related endotoxemia, which contributes to up to 50% of mortality in ICU patients [7, 8]. The mortality rate in septic AKI patients is higher than nonseptic AKI individuals [8, 9]. Endotoxemia mainly resulted from lipopolysaccharide (LPS), the major component of endotoxin released from the cell wall of Gram-negative bacteria [7]. The LPS-induced endotoxemic AKI in rodent animals is one of the most commonly used animal models to study the pathogenesis and potential treatment of endotoxemic AKI [10]. Endotoxemia-induced AKI could occur under several extremely physiologically stressful conditions, including trauma, burn, and infectious diseases [11]. Renal function is acutely and severely reduced during AKI, characterized by an increase in serum creatinine level and decrease in urine output [12]. AKI also shows the hallmark of renal tubular damage and inflammation [13–15]. LPS activates a renal inflammatory cascade, promotes the release of numerous pro-inflammatory cytokines, and results in kidney end-organ damage [16]. Thus, researchers highlight anti-inflammatory agents that may be developed into drugs for the treatment of endotoxemic AKI [17].

Evodiamine (EVO), ((+)-(S)-8,13,13b,14-tetrahydro-14-methylindolo [2′,3′:3,4] pyrido [2,1-b]quinazolin-5(7H)-one), is an important alkaloidal component extracted from* Evodia rutaecarpa*. EVO is an active ingredient of many traditional medicinal formulations, such as plant extracts of* E. rutaecarpa (Rutaceae)*, root bark of* Zanthoxylum budrunga* wall, and* Evodiae fructus* [18]. EVO has been shown to possess the properties of analgesia, antiemesis, vascular dilatation, and prevention of tumor growth and metastasis [19, 20]. In particular, EVO exhibits potent anti-inflammatory activities [21–23].

In the study, we designed experiments to evaluate the potential protective effect of EVO against LPS-induced AKI. We found that EVO had critical antioxidant and anti-inflammatory effects through inhibition of ROS/NF-*κ*B signaling and protected against LPS-induced AKI.

## 2. Materials and Methods

### 2.1. Chemicals and Reagents

LPS (Escherichia coli 055:B5), N-acetylcysteine (NAC), Evodiamine, and DCFH-DA were obtained from Sigma-Aldrich (St.Louis, MO, USA). The *β*-actin and P65 NF-*κ*B antibodies were obtained from Santa Cruz Biotechnology (Santa Cruz, CA, USA). Rat TNF-*α* and IL-1*β* ELISA kits were obtained from Thermo Fisher Scientific (Rockford, IL, USA).

### 2.2. Animals and Treatment

Male SD rats (6 -8 weeks) were purchased from the Experimental Animal Center of the First Affiliated Hospital of Xinxiang Medical University. Experiments with animals were conducted in accordance with the guidelines of the National Institutes of Health and the First Affiliated Hospital of Xinxiang Medical University. The mice were housed in plastic cages with 24 ± 2°C and 40-80% humidity with access to food and water at liberty and were kept on a 12 h light/dark cycle.

The rats were randomly allocated into the following groups (n=10):

  Control group: rats were injected with equal volume of vehicle.
  LPS group: rats were injected intraperitoneally (i.p.) with 15 mg/kg/bw of LPS in 50*μ*L PBS.
  LPS + 100 mg/kg Nerolidol group: 1 h after LPS treatment, the rats were injected i.p. with 100 mg/kg EVO.
  LPS + 200 mg/kg Nerolidol group: 1 h after LPS treatment, the rats were injected i.p. with 200 mg/kg EVO.

Experiments were terminated 24 h after LPS challenge and the blood samples and kidney tissues were collected.

### 2.3. Histological Analysis

The kidney tissues were fixed in 4% paraformaldehyde, embedded in paraffin, and sliced into 5 *μ*m sections. After staining with hematoxylin and eosin (H&E), pathological changes were observed under a light microscope (×200; Olympus, Japan). The score of histological injury was evaluated as previously reported [24].

### 2.4. Cell Culture and Treatment

The NRK-52E rat proximal tubular cell line was obtained from American Type Culture Collection (ATCC, Manassas, VA, USA). Cells were cultured in Dulbecco's modified Eagle's medium (DMEM) supplemented with 10% FBS and antibiotics (100 U/ml penicillin G, 100 mg/ml streptomycin, and 0.25 mg/ml amphotericin in an incubator with 5% CO_2_ at 37°C). Cells were cultured in serum-free DMEM with 1 *μ*g/ml LPS in the presence or absence of 10 and 20 *μ*M EVO for 24h. EVO was dissolved in DMSO as stock solution and diluted in serum-free DMEM.

### 2.5. Transfection of Plasmids

The sequence of P65 was cloned into pCMV vector. Transient transfection of plasmids was performed using Lipofectamine 2000 (Invitrogen, Carlsbad, CA, USA) according to the manufacturer's protocols. 4-6 hours after the transfection, cell growth medium was removed and incubated in media containing 5% FBS. 48 hours after the transfection, cells were incubated with 1 *μ*g/ml LPS with or without 20 *μ*M EVO for 24h. Cell viability and indicated gene expression were determined.

### 2.6. Cell Viability

The cell viability was determined by the 3-(4,5-di- methylthiazol-2-yl)-2,5-diphenyltetrazolium bromide (MTT) assay. The NRK-52E cells were seeded in 96-well plates at a density of 5 × 10^4^ cells/ml for 24 h and then were cultured in serum-free DMEM with1 *μ*g/ml LPS in the presence or absence of 10 and 20 *μ*M EVO for 24h. Thereafter, 20 *μ*l of MTT was added to each well and incubated for 4 h. After careful removal of medium, 150 *μ*l of DMSO was added. The absorbance at a wavelength of 490 nm was detected on a spectrophotometer (Bio-Rad, CA, USA).

### 2.7. Biochemical Determination

Serum and kidney homogenates were used for biochemical determination. The levels of BUN were determined using ELISA kits (MyBioSource, CA, USA) according to the manufacturer's instructions. Creatinine level was measured using colorimetric/fluorometric assay kits (BioVision, Inc., Milpitas, CA, USA) according to the manufacturer's instructions. The levels of inflammatory cytokines TNF*α* and IL-1*β* in serum and kidney homogenates were measured by ELISA kits (R&D Systems Inc., Minneapolis, MN, USA) according to the manufacturer's protocols.

### 2.8. RNA Extraction and Real-Time RT-PCR

Total RNA was isolated from the kidney by using TRIzol reagent as per the manufacturer's instructions (Life Technologies, Carlsbad, CA). Then 1 *μ*g of DNA-free total RNA was reverse-transcribed by use of a one-step RT-PCR kit (TaKaRa, Dalian, China). Reactions were performed in a 50 *μ*L SYBR GREEN PCR volume formatted in CFX96 detection systems (Bio-Rad, Hercules, CA). *β*-actin was used as an endogenous control for RNA quality and differences among samples. Fold induction was calculated according to the 2^-ΔΔCt^ values.

### 2.9. Western Blot

Total proteins were extracted using ice-cold RIPA lysis buffer (Thermo Fisher Scientific, Rockford, IL, USA) and protein concentrations were determined using a bicinchoninic acid (BCA) assay kit (Thermo Fisher Scientific, Rockford, IL, USA). The extracts were run on an SDS- PAGE gel for Western blot analysis. After electrophoresis, the proteins were electrotransferred to a polyvinylidene difluoride membrane (Millipore Corporation, MA, USA) and non-specific binding of antibodies was blocked with 5% BSA in tris-buffered saline (TBS) for 1 h at room temperature. The membranes were incubated at 4°C overnight with primary antibody in TBST. Then, the membranes were washed four times using TBST with 15 min each time. Membranes were incubated with peroxidase-conjugated IgG secondary antibody for 30 min at 37°C. After washing for four time with TBST, the immune complexes were detected using an ECL kit (Millipore Corporation, MA, USA). Target gene expression levels were normalized to *β*-actin expression.

### 2.10. ROS Level

After the treatment, cells were harvested and incubated with 10 *μ*M 2'7'-dichlorodihydrofluorescein diacetate (DCFH-DA) in serum-free DMEM for 30 min at 37°C. Analysis was performed on a flow cytometry (BD, San Jose, CA, USA). ROS level was expressed as folds vs control.

### 2.11. Statistical Analysis

Data are shown as the means ± standard error of the means (SEM). Statistical analyses were performed using GraphPad Prism software (La Jolla, CA, USA). Differences were analyzed by one-way analysis of variance, followed by Dunnett's multiple comparison test. P<0.05 was considered to be statistically significant.

## 3. Results

### 3.1. EVO Protects against LPS-Induced Renal Injury In Vivo and In Vitro

**Figure 1:**
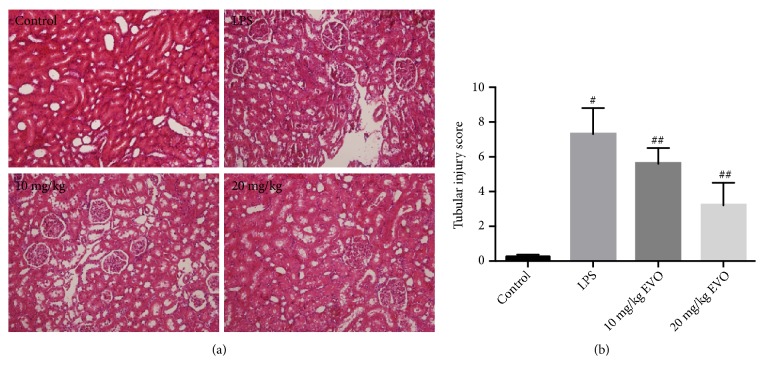
*Effects of EVO on pathological injury of kidney in rats treated by LPS.* Rats were intraperitoneally (i.p.) injected with EVO (10 and 20 mg/kg) 1 h after LPS treatment. Hematoxylin and eosin (HE) staining was conducted to evaluate histological injury of kidney tissues. Representative images of HE staining were shown (a). The score of tubular injury was calculated (b). #p<0.05 vs control group. ##p<0.05 vs LPS group.

**Figure 2:**
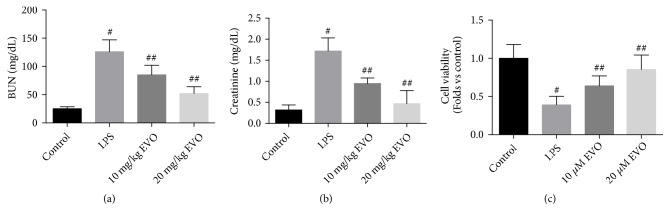
*Effects of EVO on functional injury of kidney in rats and cell viability in NRK-52E cells treated by LPS.* Rats were intraperitoneally (i.p.) injected with EVO (10 and 20 mg/kg) 1 h after LPS treatment. Kidney function was evaluated by the determination of blood urea nitrogen (BUN) (a) and creatinine (b) levels. NRK-52E cells were treated with 1 *μ*g/ml LPS in the presence or absence of 10 and 20 *μ*M EVO for 24h. Cell viability was detected by MTT assay (c). #p<0.05 vs control group. ##p<0.05 vs LPS group.

LPS-induced rat model of endotoxemic AKI was established in our study. We showed that the injection of LPS induced edema of renal tubular epithelial cells, tubular dilation, and distortion in kidneys of rats. LPS-resulting pathological injuries were significantly ameliorated by the administration of EVO (Figure 1(a)). The tubular injury score in LPS group was significantly reduced by EVO (Figure 1(b)). In addition, LPS induced a marked increase in the levels of blood urea nitrogen (BUN) and creatinine (Figures 2(a) and 2(b)). The treatment of EVO notably reduced the levels of BUN and creatinine (Figures 2(a) and 2(b)). Moreover, NRK-52E cells were exposed to LPS to induce cytotoxicity. In Figure 2(c), we showed that LPS resulted in a significant decrease of cell viability in NRK-52E cells. In the presence of EVO, the reduction of cell viability induced by LPS was notably inhibited (Figure 2(c)). The data demonstrated that EVO protected against LPS-induced AKI in vivo and in vitro.

### 3.2. EVO Inhibits LPS-Induced Inflammation In Vivo and In Vitro

**Figure 3:**
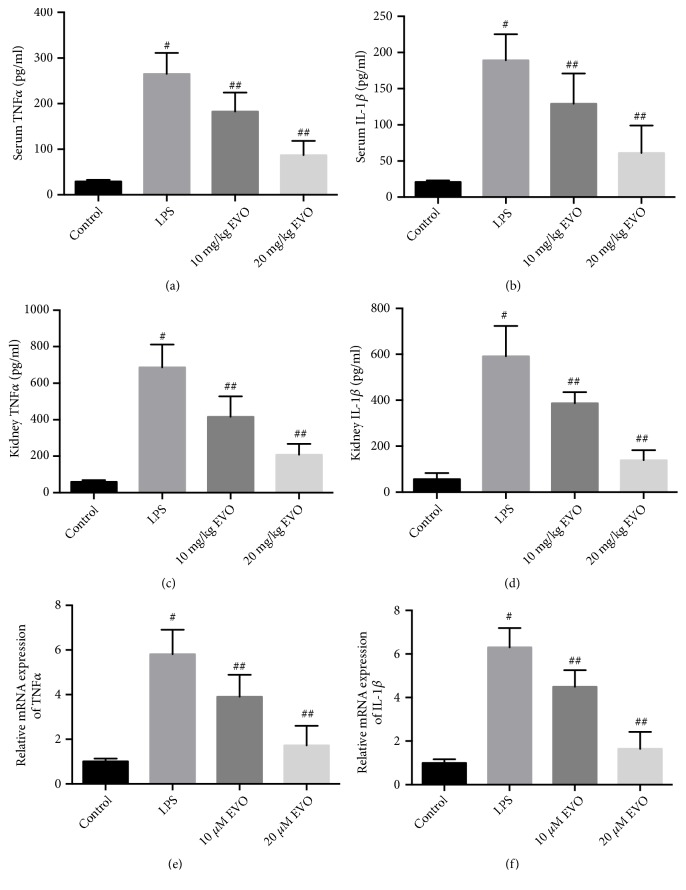
*Effects of EVO on proinflammatory cytokines in rats and in NRK-52E cells treated by LPS. *Rats were intraperitoneally (i.p.) injected with EVO (10 and 20 mg/kg) 1 h after LPS treatment. Inflammation was evaluated by the determination of serum (a and b) and tissue (c and d) levels of proinflammatory cytokines, including TNF*α* (a and c) and IL-1*β* (b and d). NRK-52E cells were treated with 1 *μ*g/ml LPS in the presence or absence of 10 and 20 *μ*M EVO for 24h. Relative mRNA expression of TNF*α* (e) and IL-1*β* (f) was measured. #p<0.05 vs control group. ##p<0.05 vs LPS group.

*The Effect of EVO on Inflammation under the Condition of LPS-Induced AKI. *In Figures 2(a), 2(b), 2(c), and 2(d), we showed that serum and kidney levels of inflammatory cytokines TNF*α* and IL-1*β* were significantly increased. The treatment of EVO induced a marked reduction of the levels of TNF*α* and IL-1*β* in LPS-treated rats (Figures 3(a), 3(b), 3(c), and 3(d)). In addition, the mRNA expression of TNF*α* and IL-1*β* was notably increased by LPS in NRK-52E cells (Figures 3(e) and 3(f)). LPS-induced increase of TNF*α* and IL-1*β* levels was blocked by EVO treatment (Figures 3(e) and 3(f)). The data demonstrated that EVO exhibited anti-inflammatory effects against LPS-induced AKI.

### 3.3. Inhibition of NF-*κ*B Expression Is Involved in the Protective Effects of EVO

**Figure 4:**
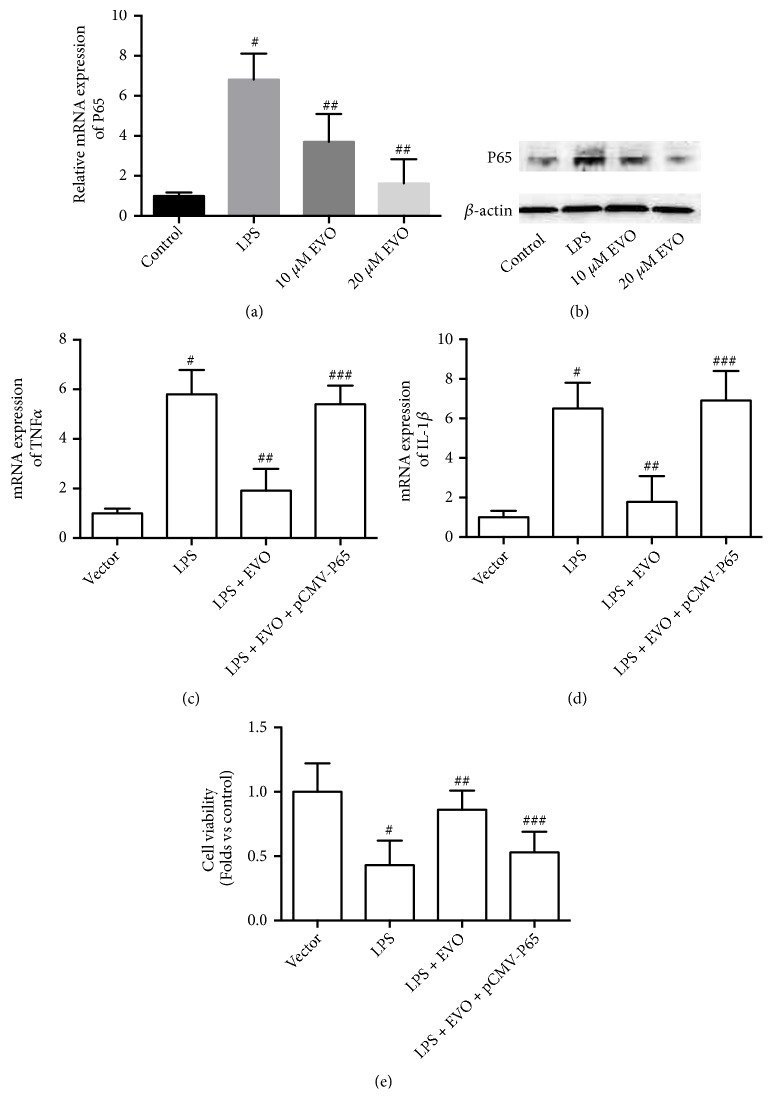
*Role of NF-κB in EVO-induced protective effect against LPS-induced cytotoxicity in NRK-52E cells.* NRK-52E cells were treated with 1 *μ*g/ml LPS in the presence or absence of 10 and 20 *μ*M EVO for 24h. Relative mRNA (a) and protein (b) expression of P65 NF-*κ*B were measured. NRK-52E cells were transfected with pCMV vector or pCMV-P65 and exposed to 1 *μ*g/ml LPS with or without 20 *μ*M EVO for 24h. Relative mRNA expression of TNF*α* (c) and IL-1*β* (d) was measured. Cell viability was detected by MTT assay (e). #p<0.05 vs control group. ##p<0.05 vs LPS group. ###p<0.05 vs LPS+EVO group.

To explore the mechanism of EVO-induced anti-inflammatory effects, we examined the expression of P65 NF-*κ*B. In Figures 4(a) and 4(b), we showed that LPS resulted in a significant increase of P65 NF-*κ*B mRNA and protein expression in NRK-52E cells. This increase of P65 NF-*κ*B expression was markedly inhibited by EVO (Figures 4(a) and 4(b)). To test whether the reduction of P65 NF-*κ*B expression was involved in the protective effects of EVO against AKI, the expression of P65 NF-*κ*B was upregulated in NRK-52E cells using pCMV-P65 NF-*κ*B. As shown in Figures 4(c) and 4(d), EVO-induced reduction of TNF*α* and IL-1*β* expression was blocked by overexpression of P65 NF-*κ*B. Moreover, the increase of cell viability in LPS-treated cells induced by EVO was remarkably suppressed by overexpression of P65 NF-*κ*B (Figure 4(e)). The data demonstrated that downregulation of P65 NF-*κ*B was responsible for the anti-inflammatory effects of EVO and was involved in EVO-induced protective effects against LPS-induced AKI.

### 3.4. Antioxidant Effect Is Involved in the Protective Effects of EVO

**Figure 5:**
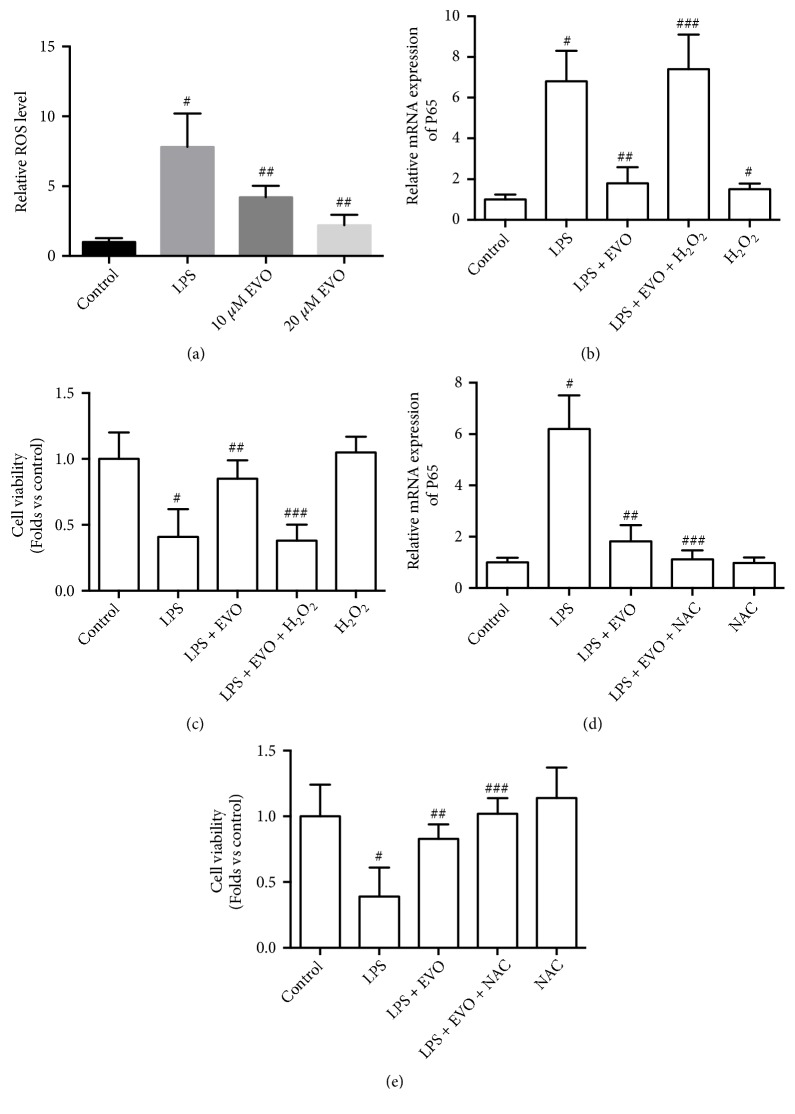
*Role of ROS in EVO-induced protective effect against LPS-induced cytotoxicity in NRK-52E cells.* NRK-52E cells were treated with 1 *μ*g/ml LPS in the presence or absence of 10 and 20 *μ*M EVO for 24h. Relative mRNA (a) and protein (b) expression of P65 NF-*κ*B was measured. NRK-52E cells were transfected with pCMV vector or pCMV-P65 and exposed to 1 *μ*g/ml LPS with or without 20 *μ*M EVO for 24h. Relative mRNA expression of TNF*α* (c) and IL-1*β* (d) was measured. Cell viability was detected by MTT assay (e). #p<0.05 vs control group. ##p<0.05 vs LPS group. ###p<0.05 vs LPS+EVO group.

In the next step, we explored the mechanism of EVO-induced inhibition of P65 NF-*κ*B expression. The ROS level was examined and the results showed that LPS resulted in a significant increase in ROS generation in NRK-52E cells (Figure 5(a)). LPS-resulting ROS production was suppressed by EVO in a concentration-dependent manner (Figure 5(a)). This finding indicated that EVO played an antioxidant role under the condition of LPS-induced cytotoxicity in NRK-52E cells. Next, we tested whether the antioxidant role was involved in EVO-induced anti-inflammatory effects and protective effects against LPS-induced cytotoxicity. Hydrogen peroxide (H_2_O_2_) treatment increased P65 NF-*κ*B expression (Figure 5(b)). H_2_O_2_ suppressed EVO-induced decrease of P65 NF-*κ*B expression in LPS-treated NRK-52E cells (Figure 5(b)). In addition, EVO-induced increase of cell viability in LPS-treated NRK-52E cells was inhibited by H_2_O_2_ treatment (Figure 5(c)). Moreover, the antioxidant NAC significantly promoted EVO-induced decrease of P65 NF-*κ*B expression (Figure 5(d)) and increase of cell viability (Figure 5(e)) in LPS-treated NRK-52E cells. The data demonstrated that the antioxidant activity was involved in the anti-inflammatory effects and protective effects of EVO against LPS-induced cytotoxicity.

## 4. Discussion

LPS is the most common agent that is used to establish endotoxemic AKI animal model and induce cytotoxicity in renal cells. In the current study, we examined the effects of EVO on LPS-induced AKI in rats and LPS-induced in NRK-52E rat proximal tubular cells. We found that EVO ameliorated the histological injury of kidney tissues and improved the function of kidney, as reflected by decrease of BUN and creatinine levels, indicating that EVO protected against LPS-induced AKI. EVO also exhibited cytoprotective effects against LPS in NRK-52E cells.

The kidney is bound with high flow of blood and is sensitive to systemic inflammation. In turn, cytokines and chemokines can be synthesized within the tubular epithelium and released to the circulation [25]. Thereafter, the kidney is easily subject to inflammatory injury [26, 27], resulting in renal dysfunction [28]. EVO has been reported to exhibit anti-inflammatory activities. For example, EVO was found to inhibit nitric oxide and PGE2 synthesis from lipopolysaccharide or IFN-*γ*-stimulated RAW264.7 cells [22, 23, 29]. EVO may regulate inducible nitric oxide synthase via affecting the expression of inflammatory cytokines, such as TNF*α* [30]. Fan et al. found that EVO inhibited zymosan-induced production of IL-6, TNF*α*, and IL-1*β* at both mRNA and protein levels at 6 h in RAW264.7 cells [31]. However, it was not reported whether EVO had an anti-inflammatory effect in kidney. In the current study, we found that EVO significantly inhibited LPS-induced increase of serum and kidney levels of TNF*α* and IL-1*β*. EVO inhibited LPS-induced increase of P65 NF-*κ*B expression and overexpression of P65 NF-*κ*B markedly reduced the anti-inflammatory activity and the protective effects of EVO against LPS-induced cytotoxicity in kidney cells. We suggest that the anti-inflammatory activity may participate in the protective effects of EVO against LPS-induced AKI.

The NF-*κ*B signal pathway lies in the center of inflammatory and immune response [32, 33]. ROS has been reported to activate NF-*κ*B through the classical IKK-dependent pathway and induces a positive feedback mechanism associated with inflammation and kidney injury [34–36]. In the current study, we also tested the role of ROS regulation in the protective effect of EVO. We showed that EVO played an antioxidant role in the protection against LPS-induced cytotoxicity. Addition of oxidant H_2_O_2_ could reverse, but NAC could promote EVO-induced inhibition of P65 NF-*κ*B expression and increase of cell viability in LPS-treated cells. The results suggested that EVO had the anti-inflammatory and renal protective effects via its antioxidant role.

In conclusion, we showed that EVO had crucial renal protective effects against LPS-induced AKI and cytotoxicity through the antioxidant activities and thus the inhibition of inflammation. Our data highlight EVO as a potential candidate for the development of new strategies for the treatment of AKI.

## Data Availability

The data used to support the findings of this study are available from the corresponding author upon request.
